# *Parabacteroides goldsteinii* mitigates the lipopolysaccharide-induced inflammatory response in IPEC-J2 cells

**DOI:** 10.3389/fvets.2025.1577126

**Published:** 2025-05-07

**Authors:** Junjie Zhang, Anjian Li, Yang He, Taozong Guo, Yixuan Ge, Hongbin Pan, Cuilian Dong

**Affiliations:** ^1^Yunnan Provincial Key Laboratory of Animal Nutrition and Feed Science, Faculty of Animal Science and Technology, Yunnan Agricultural University, Kunming, China; ^2^School of Agriculture and Life Sciences, Kunming University, Kunming, China

**Keywords:** *Parabacteroides goldsteinii*, IPEC-J2 cell, lipopolysaccharide, anti-inflammatory mechanism, PI3K-Akt pathway

## Abstract

**Introduction:**

The misuse of antibiotics threatens animal health and food safety and limits the development of livestock and poultry farming. In animal husbandry, probiotics can be used as an alternative to antibiotics because of their environmental protection and high efficiency. *Parabacteroides goldsteinii* (PG) has positive anti-inflammatory effects in mice; however, its role in other animals remains unknown.

**Methods:**

The results showed that cell viability with the PG supernatant was highest at a 20-fold dilution ratio. Exposure of IPEC-J2 cells to LPS (1 μg/mL) significantly increased IL-6 and IL-8 concentrations, which triggered an inflammatory response. PG attenuated the LPS-induced inflammatory response by inhibiting the release of pro-inflammatory cytokines (IL-6) and promoting the release of anti-inflammatory cytokines (IL-10). Transcriptome analysis showed that the average number of clean reads in the LPS and LPS-PG groups was 39,311,061 and 38,085,237, respectively. In total, 2,126 differentially expressed genes (DEGs) were identified between the LPS and LPS-PG groups, including 36 up-regulated and 2090 down-regulated genes. Some DEGs, such as IL-6R and NF-кB, are related to inflammation. GO analysis was used to annotate the functions of the DEGs, and the results showed that biological regulation, cellular anatomical entity, and binding were dominant. KEGG enrichment analysis showed that the DEGs were significantly enriched in the PI3K-AKT signaling pathway, protein export pathway, and antigen processing and presentation pathway.

**Discussion:**

These results indicate that *P. goldsteinii* is a promising probiotic for maintaining and improving intestinal health in piglets.

## 1 Introduction

With the rising global demand for animal-derived products, intensive livestock farming has become essential for maximizing production efficiency (1). However, this has led to increased disease risks, prompting the widespread use of antibiotics in animal husbandry. However, long-term abuse of antibiotics reduces disease resistance in animals, increases resistance among pathogenic bacteria, and leads to drug residues in animal products (2). These factors endanger food safety and limit the development of livestock and poultry farming. As several antibiotics are forbidden in livestock feed, it is important to identify feed additives that can replace antibiotics. Therefore, developing environmentally friendly and safe antibiotic alternatives is a priority in livestock farming.

Probiotics are considered green, safe, and can effectively maintain the microbial balance of the host gut and improve immune function (3). They are considered viable antibiotic alternatives because they are non-toxic, leave no residues, do not contribute to antibiotic resistance, are cost-effective, promote animal health, and are environmentally sustainable (4). Furthermore, probiotic metabolites can have various beneficial effects on the host. For example, short-chain fatty acids (5), indoles (6), vitamins (7), and polyphenols (8) play protective roles in intestinal barrier function. Studies have shown that probiotics such as *Bacillus subtilis* (9), *Clostridium butyricum* (10), and *Lactobacillus* spp. (11) effectively improve growth performance, digestive enzyme activity, and the intestinal morphology of livestock. Previous studies on probiotics have primarily focused on aerobic and facultative anaerobic bacteria. In contrast, anaerobic bacteria such as *Parabacteroides goldsteinii* (PG) have not been widely investigated and are thus not commonly used in livestock and poultry farming. PG helps to reduce inflammation in mice (12), ameliorates chronic obstructive pulmonary disease (COPD) induced by cigarette smoke in mice, reduces intestinal inflammation, and enhances mitochondrial and ribosome activity in colon cells (43). Thus, PG could be a promising next-generation probiotic.

In this study, we established an LPS-induced porcine small intestinal epithelial cell line (IPEC-J2) to explore the effects of PG extracts by comparing cytokine levels and analyzing its mechanism of action in IPEC-J2 cells. These results help to establish a theoretical basis for the use of PG in the pig farming industry.

## 2 Methods

### 2.1 Culture and preparation of PG supernatant

PG was obtained from the Microbe Division, Institute of Physical and Chemical Research (Japan) and cultured in Gifu Anaerobic Medium (GAM) using an LAI-3 T-N anaerobic incubator (Shanghai Longyue Instrument Equipment Co., Ltd., China). The conditions and operation steps of the Institute of Physical and Chemical Research (Japan) were strictly followed. When the OD_600_ reached 0.591 and there were 7.9 × 10^7^ CFUs, the cultivation was terminated. DNA extraction was performed immediately using 1 mL of the bacterial solution. The supernatant was collected via centrifugation at 5,000 × g for 20 min and stored at −80°C.

### 2.2 IPEC-J2 cell culture

IPEC-J2 cells were cultured in high-glucose DMEM containing 10% FBS, 10,000 U/mL penicillin, and 10,000 μg/mL streptomycin in a 37°C and 5% CO_2_ incubator (13). The medium was changed every 2 days. When a confluence of ~80% was reached, cells were trypsinized and seeded in six-well cell culture plates (3 × 10^5^ cells/well). After overnight culture, cells were starved for 6 h in Arg-free DMEM-F12. The PG-derived supernatant was diluted 20, 100, 500, 2,500, and 12,500-fold and added to the culture medium for probiotic functional analysis.

### 2.3 Cell viability assay

After overnight culture in 96-well plates (1 × 10^5^ cells/well, 100 μL medium/well), cells were treated with different concentrations of LPS and dilutions of the PG bacterial solution supernatant for 24 h. Thiazolyl blue tetrazolium bromide (MTT; 10 μL) was added to each well, and the cells were incubated at 37°C and 5% CO_2_ for 4 h. Dimethyl sulfoxide (DMSO; 100 μL) was added, and the mixture was incubated at 37°C for 10 min. Finally, the absorbance was evaluated at 540 nm after shaking for 10 min (14).

### 2.4 Construction of the experimental model

IPEC-J2 cells were cultured as described above, and when 80% confluence was reached, they were treated with LPS (0.2, 1, 5, 25, and 125 μg/mL) derived from *Escherichia coli* for 24 h.

After the inflammation model were established, IPEC-J2 cells were administered LPS (1 μg/mL), and co-incubated with PG supernatant (0- or 20-fold dilution). After 24 h, cells were harvested via centrifugation.

### 2.5 RNA-seq

Total RNA of IPEC-J2 cell was extracted from the supernatant of logarithmically growing IPEC-J2 cells using TRIzol (Invitrogen, United States) according its introduction. The concentrations of RNA were detected by NanoDrop 2,000 spectrophotometer (Thermo Scientific, United States), and its integrity was determined by the RNA Nano 6,000 Assay Kit (Agilent Technologies, United States) in the Agilent Bioanalyzer 2,100 system (Agilent Technologies, United States). A cDNA library was constructed using the Illumina VAHTS Universal V10 RNA-seq Library Prep Kit (MGI Tech, China) following manufacturer’s protocols. Poly-A tailed mRNA was enriched using oligo (dT)-coated magnetic beads, and the mRNA was fragmented via ultrasound. The fragmented mRNA was used as a template, and random oligonucleotides were used as primers to synthesize the first strand of cDNA using the M-MuLV reverse transcriptase system. The RNA strand was degraded using RNase H, and the second cDNA strand was synthesized using DNA polymerase I and dNTPs. The purified double-stranded cDNA was end-repaired and A-tailed, and the sequencing joints were connected. cDNA (~200 bp) was obtained using AMPure XP beads, amplified using, and the products were then purified using AMPure XP beads. Double-ended sequencing was performed using an Illumina NovaSeq 6000 sequencing system in Lingen Biotechnology Ltd., Shanghai, China.

### 2.6 Analysis of interleukin (IL)-6, IL-8, and IL-10 levels

IL-6, IL-8, and IL-8 concentrations were measured using the Interleukin-6, Interleukin-8, and Interleukin-10 ELISA Assay Kits (Nanjing Jiancheng Bioengineering Institute, Nanjing, China).

### 2.7 RNA-seq data analysis

The raw sequencing reads were filtered for low-quality sequences (such as adapter sequences, N > 10% and junction sequences) by Trimmomatic (SLIDINGWINDOW:4:15 MINLEN:75) (version 0.36)^1^ to ensure the reliability of the sequencing results (15). The base composition and quality of the clean reads were then analyzed. Then HISAT2^2^ software was used to align clean reads to the reference genome. The overall quality of the RNA-seq data was evaluated based on the coverage, ribosomal reads, and comparison of the regional statistics. The Q20 (the proportion of bases with a phred score greater than 20), Q30 The Q20 (the proportion of bases with a phred score greater than30) and GC content were used to determine the RNA-Seq quality.

To account for the effects of sequencing coverage and gene length, the number of read counts was normalized using the fragments per kilobase of exon model per million mapped reads (FPKM) method and used as the input for subsequent analysis (16). Principal component analysis (PCA) was used to evaluate the reproducibility of the samples and exclude outliers. Differentially expressed genes (DEGs) were analyzed using edgeR. Genes with a log2 fold change (|log2FC|) ≥ 1 and a false-discovery rate (FDR)-adjusted *p*-value (padj) < 0.05 between the LPS and LPS-PG groups were considered differentially expressed.

The Gene Ontology (GO) database^3^ was used to investigate the differences in biological functions of the DEGs. The Kyoto Encyclopedia of Genes and Genomes (KEGG) database^4^ was used to investigate signaling and metabolic pathways associated with the DEGs (17).

### 2.8 DEG verification using qPCR

To validate the RNA-seq results, eight DEGs were randomly selected and qPCR was performed using the SYBR Green Supermix kit (Bio-Rad, United States) to verify the expression of DEGs in the LPS and LPS-PG groups. Primers were designed using the NCBI Primer BLAST tool (Table 1).^5^ cDNA was synthesized using the HiScript II 1st Strand cDNA Synthesis Kit (Vazyme, Nanjing, China). The PCR reaction condition was as follows: 95°C for 30 s, s, 40 cycles of 95°C for 5 s, 56°C for 30 s, and 72°C for 1 min. The *β*-actin gene was used as an internal reference, and the target mRAN expression was calculated using the 2^–ΔΔ^CT method.

**Table 1:** Primer sequences.

Primer	Sequences (5′–3′)
ABCA1 forward	TTGCTGAAGATGCTGGAGGG
ABCA1 reverse	CCAGCCCTTCACTTGGTAGG
CPB2 forward	TGGGGTCTGGCATCATAGGA
CPB2 reverse	GCCAACGCTGTGTGCAATTA
HOXB4 forward	CCTGGATGCGCAAAGTTCAC
HOXB4 reverse	GCGAGTTTATAGCGGGGACA
CD9 forward	TGGCCTGGGATCTGGACATA
CD9 reverse	GACGGCAACAGATGAGGTCA
CLDN2 forward	TGTCAGCGCAGAACAGGAAT
CLDN2 reverse	GCTCTGGCAAGGAAGTCACT
PNN forward	GTTGCATCCCACCTTTGCAG
PNN reverse	CCGGTCTCCACACTTCCTTC
IGF1R forward	AGGGGAAGAGAGGCAGTAGG
IGF1R reverse	GAGTGCAGAGAAACCGTCCA
β-actin forward	GGATGCAGAAGGAGATCACG
β-actin reverse	ATCTGCTGGAAGGTGGACAG

### 2.9 Statistical analysis

Data are expressed as the mean ± SEM. SPSS 27.0 was used to conduct one-way ANOVA. Duncan’s test was used to determine significance at *p* < 0.05. Twelve cell samples were used for RNA-Seq. FPKM was used to detect DEGs between the LPS and LPS-PG groups.

## 3 Results

### 3.1 Effect of the PG supernatant on IPEC-J2 cell viability

**Figure 1:**
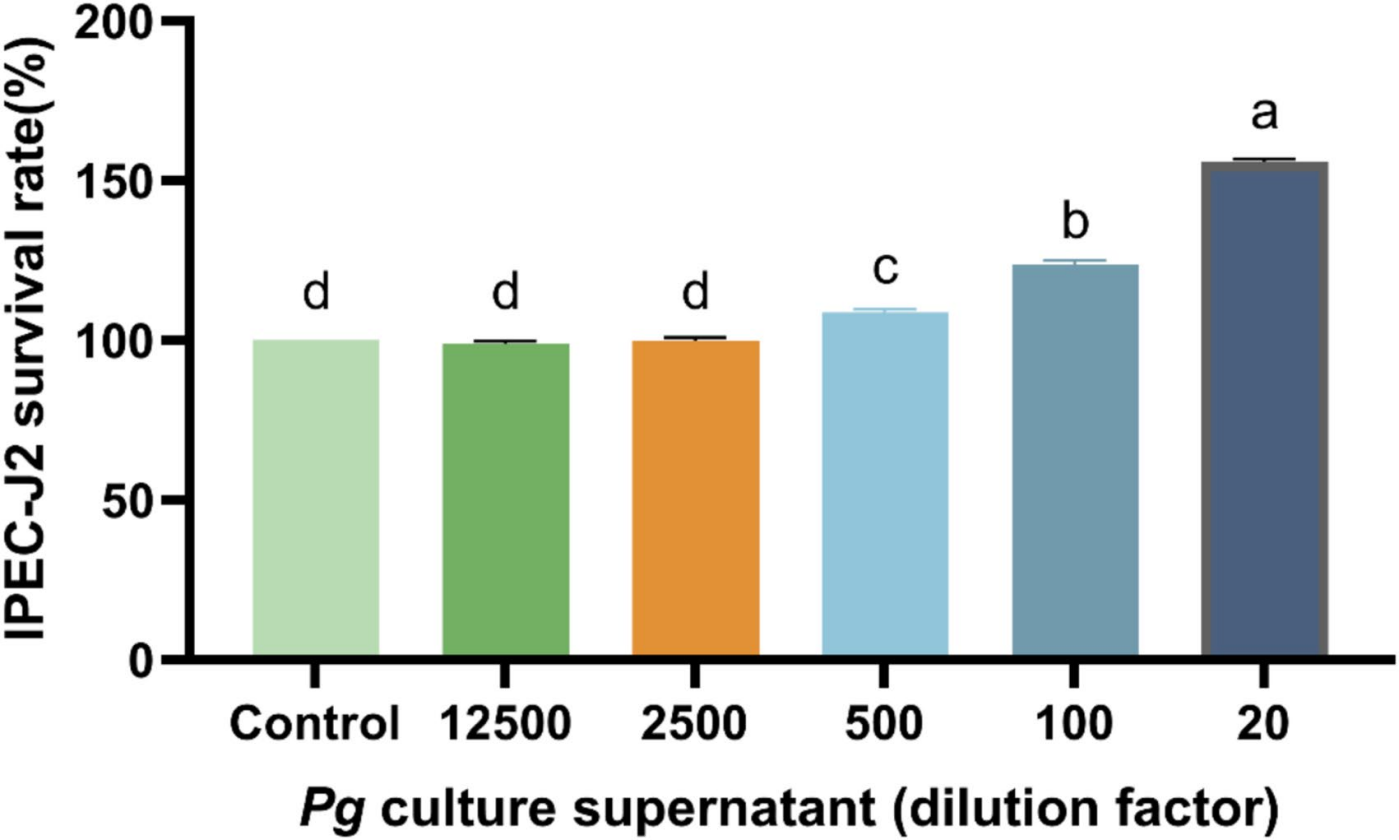
Effect of PG culture supernatant on IPEC-J2 cell viability. IPEC-J2 cells were cultured in 96-well plates and treated with various dilution factor of PG culture supernatant (ranging from 20 to 12,500) for 24 h. The results, presented as the mean ± standard error of the mean (SEM). Values with different lowercase letters indicate significant differences.

Cell viability was significantly higher (*p* < 0.05) with 500-, 100-, and 20-fold dilutions of PG supernatant than in the control group (Figure 1). Overall, cell-viability was highest with the 20-fold PG dilution, and consequently, this was used for subsequent experiments.

### 3.2 Effect of LPS on IL-6 and IL-8 secretion from IPEC-J2 cells

**Figure 2:**
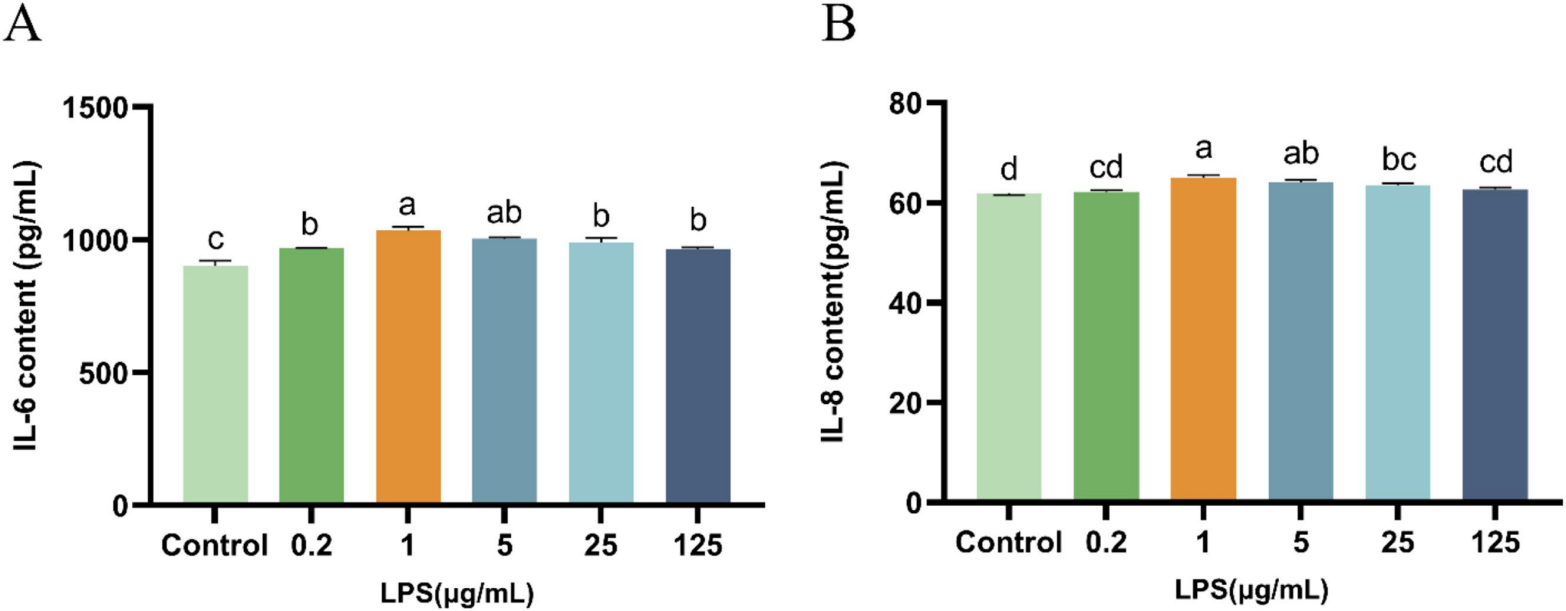
Effect of LPS on IL-6 and IL-8 secretion from IPEC-J2 cells. **(A)** IL-6. **(B)** IL-8. IPEC-J2 cells were cultured 96-well plates and treated with LPS (0.2, 1, 5, 25, and 125 μg/mL). The results, presented as the mean ± standard error of the mean (SEM). Values with different lowercase letters indicate significant differences.

Compared with the control group, LPS (0.2, 1, 5, 25, or 125 μg/mL) induced a significant increase (*p* < 0.05) in IL-6 and IL-8 levels (Figure 2). Specifically, 1 μg/mL of LPS induced the highest IL-6 and IL-8 levels, and consequently, this LPS concentration was used to construct the IPEC-J2 cellular inflammatory model.

### 3.3 Effect of PG supernatant on IL-6, IL-8, and IL-10 levels in LPS-induced IPEC-J2 cells

LPS induced a significant increase (*p* < 0.05) in IL-6 and IL-8 levels and a decrease (*p* < 0.05) in IL-10 levels in IPEC-J2 cells (Table 2). However, IL-6 and IL-8 levels were significantly decreased in the presence of PG supernatant, whereas IL-10 levels were significantly increased (p < 0.05) in LPS-induced IPEC-J2 cells (Table 2).

**Table 2:** The effect of PG culture supernatant on LPS induction of IPEC-J2 cell levels of IL-6, IL-8, and IL-10.

Items	IL-6	IL-8	IL-10
Control	7.55 ± 1.87^b^	8.57 ± 1.74^a^	21.90 ± 1.51^b^
LPS	16.85 ± 2.72^a^	10.08 ± 1.71^a^	16.49 ± 0.79^c^
LPS-PG	8.88 ± 1.57^b^	13.81 ± 0.66^a^	41.53 ± 1.14^a^

Values with different lowercase letters indicate significant differences.

### 3.4 RNA-seq analysis

To investigate the molecular mechanism by which the PG supernatant alleviates LPS-induced inflammation in IPEC-J2 cells, RNA-Seq of the LPS and LPS-PG groups was conducted.

The LPS and LPS-PG groups had an average of 39,311,061 and 38,085,237 clean reads, respectively. The sequencing quality of the Q20 (error rate < 1%) and Q30 (error rate< 0.1%) samples was 98.28–99.23% and 94.26–97.19%, respectively. The sequencing data from the samples were aligned to the pig reference genome, with an average of 93.18% of the reads mapped to the reference genome (Table 3).

**Table 3:** Summary of the transcriptome sequencing data.

Sample	Raw reads	Raw data (bp)	Clean reads	Clean reads (%)	Clean data (bp)	Q20(%)	Q30(%)	Mapped reads	Mapped rate (%)
LPS1	37,798,578	5,669,786,700	37,185,852	98.38	5,465,835,456	99.23	97.19	34,415,506	92.55
LPS2	37,606,606	5,640,990,900	28,214,970	75.03	3,965,319,567	98.45	95.76	25,610,728	90.77
LPS3	38,927,368	5,839,105,200	37,559,160	96.48	5,493,889,513	99.11	96.79	34,832,364	92.74
LPS4	49,243,794	7,386,569,100	48,410,310	98.31	7,162,424,527	98.94	96.27	44,745,649	92.43
LPS5	40,668,970	6,100,345,500	39,003,908	95.91	5,752,563,250	99.06	96.55	36,219,028	92.86
LPS6	47,153,036	7,072,955,400	45,492,170	96.48	6,721,215,524	99.15	96.85	42,125,749	92.6
LPS-PG1	38,020,976	5,703,146,400	36,009,122	94.71	5,317,997,376	98.28	94.26	33,974,606	94.35
LPS-PG2	36,984,360	5,547,654,000	35,595,180	96.24	5,277,916,928	98.78	95.89	33,377,600	93.77
LPS-PG3	38,337,454	5,750,618,100	37,079,840	96.72	5,475,150,140	99.11	96.75	34,847,633	93.98
LPS-PG4	37,824,340	5,673,651,000	36,454,494	96.38	5,388,054,267	99.15	96.81	34,150,569	93.68
LPS-PG5	38,062,214	5,709,332,100	36,966,004	97.12	5,448,263,741	98.81	95.89	34,862,638	94.31
LPS-PG6	47,982,326	7,197,348,900	46,406,784	96.72	6,777,026,598	98.81	96.1	43,687,346	94.14

### 3.5 DEG analysis

**Figure 3:**
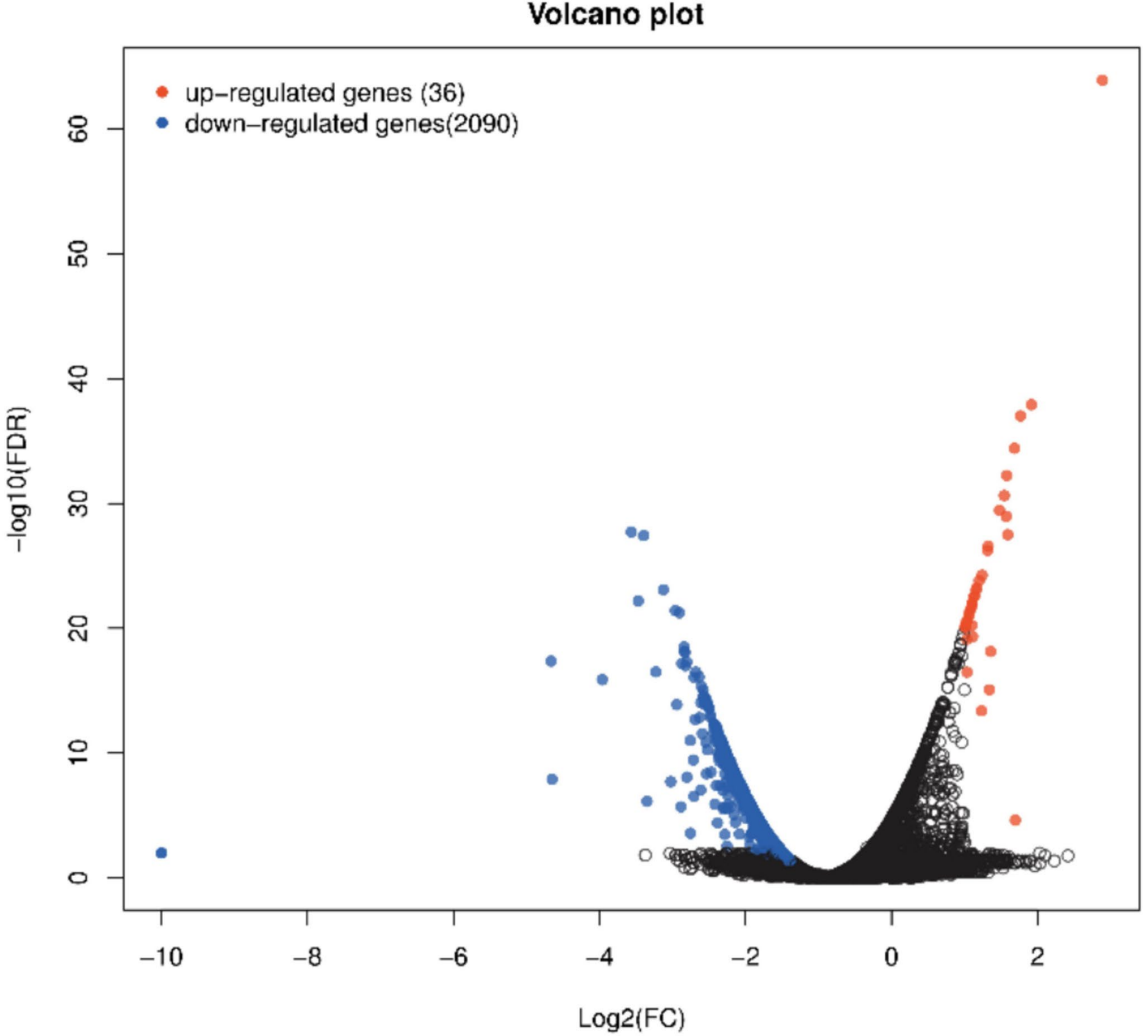
Volcano plot of significant genes from DEseq analysis of *n* = 6 LPS vs. *n* = 6 LPS-PG. Red dots show significant genes defined by |logFC| ≥ 1, FDR ≤ 0.05. Blue dots show significantly downregulated genes defined by |logFC| ≥ 1, FDR ≤ 0.05.

The LPS and LPS PG groups were analyzed, and 2,126 significantly differentially expressed genes (DEGs) were identified, including 36 upregulated genes and 2090 down-regulated genes (Figure 3). The DEGs included IL-6R, NF-κB1, IGF1R, and ABCA1, which are associated with cellular inflammation.

### 3.6 GO enrichment analysis

**Figure 4:**
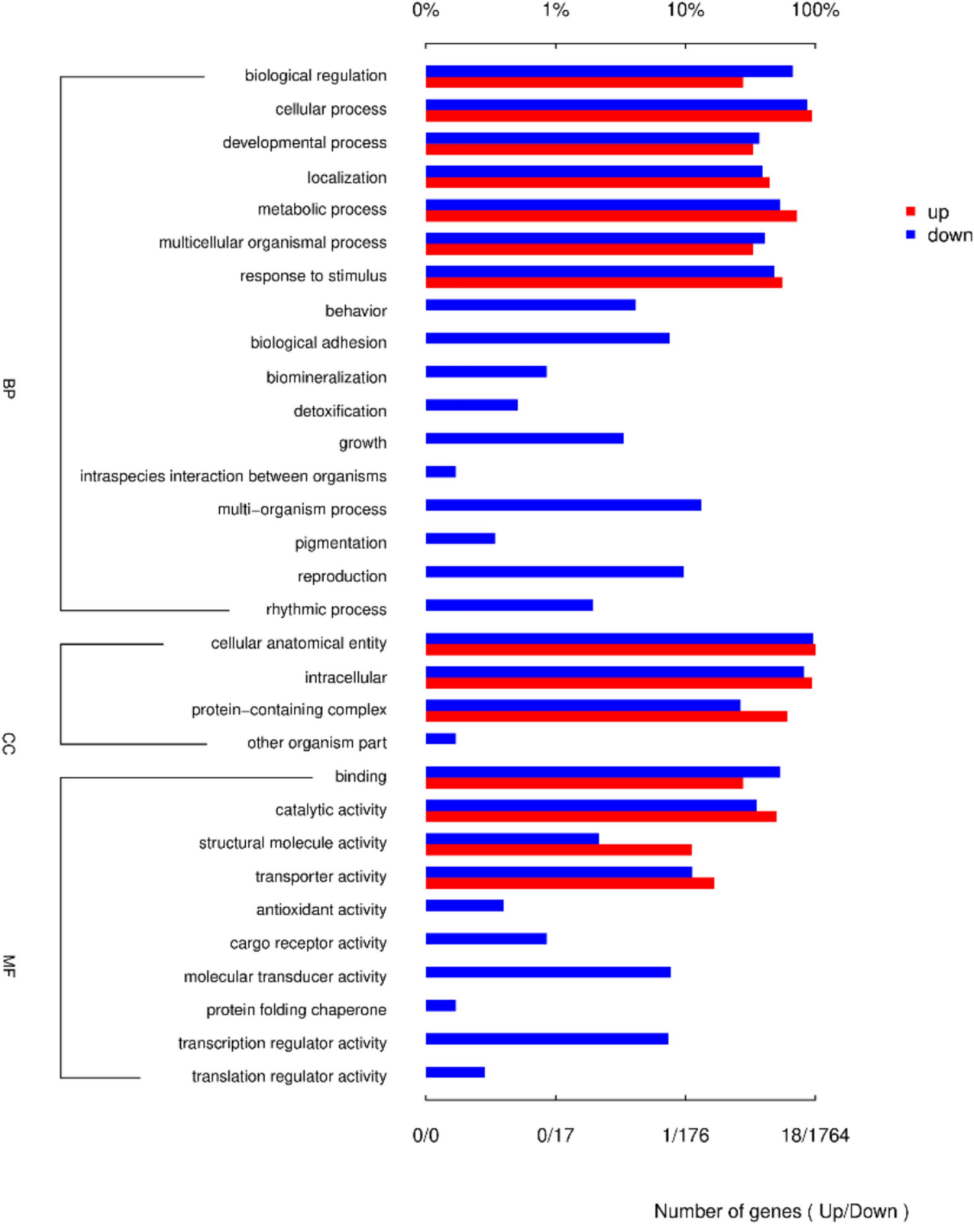
Column chart of the 30 GO terms most enriched with DEGs between LPS and LPS-PG.

GO enrichment analysis identified biological processes (BP), cellular components (CC), and molecular functions (MF). Biological regulation, cellular process, and developmental process were the most enriched BP terms; cellular anatomical entity, intracellular, and protein-containing complexes were the most enriched CC terms; and binding, catalytic activity, and structural molecule activity were the most enriched MF terms (Figure 4).

### 3.7 KEGG pathway analysis

**Figure 5:**
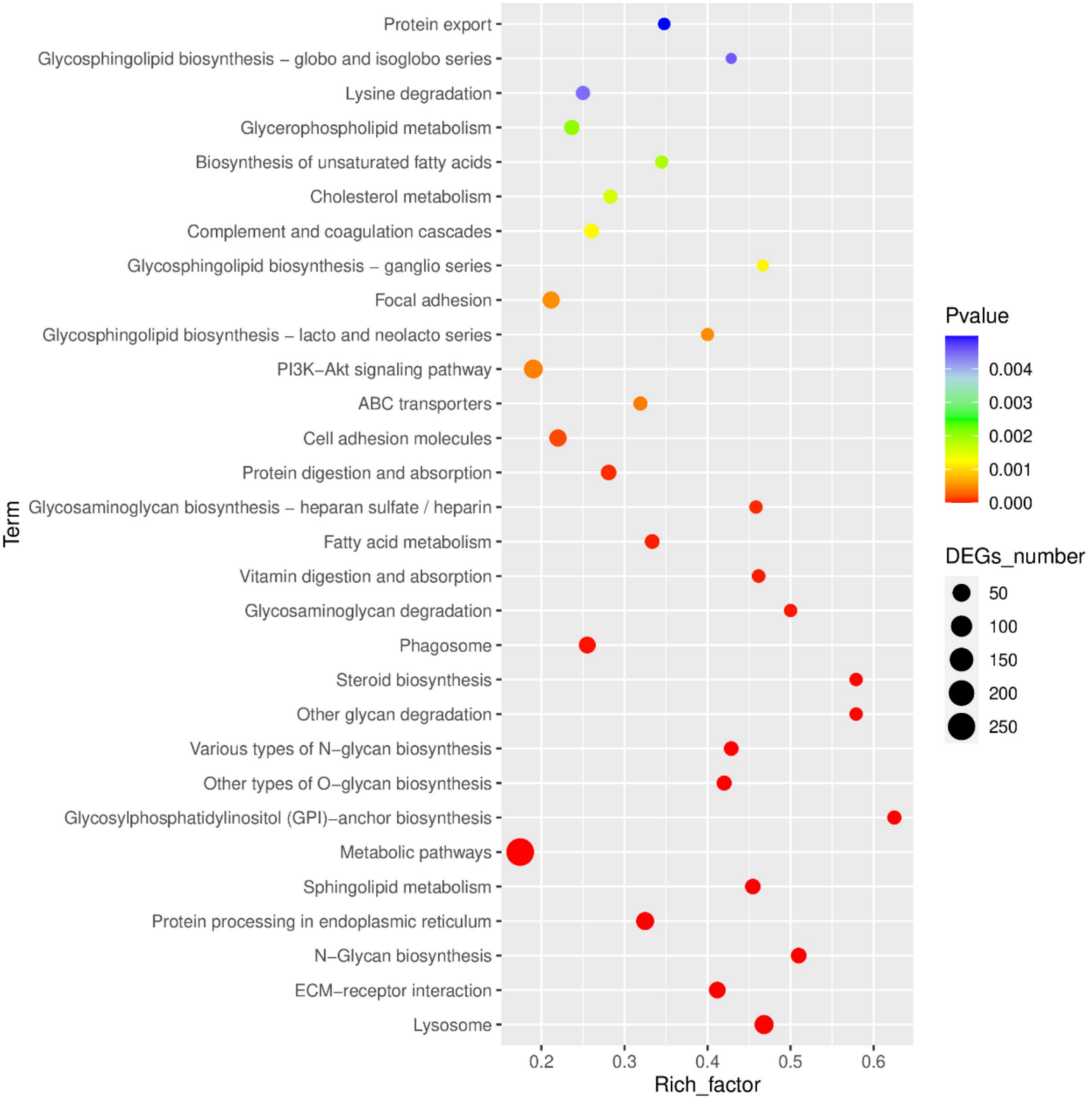
Bubble diagram of the 30 KEGG pathways most enriched with DEGs between LPS and LPS-PG.

Kegg pathway analysis identified the top 30 most significantly enriched pathways, which included the PI3K-Akt signaling pathway, protein export, antigen processing and presentation, lysosome, ECM-receptor interaction, N-glycan biosynthesis, protein processing in endoplasmic reticulum, sphingolipid metabolism, metabolic pathways, glycosylphosphatidylinositol (GPI)-anchor biosynthesis, other types of O-glycan biosynthesis, various types of N-glycan biosynthesis, other glycan degradation, steroid biosynthesis, phagosome, glycosaminoglycan degradation, vitamin digestion and absorption, fatty acid metabolism, glycosaminoglycan biosynthesis—heparan sulfate/heparin, protein digestion and absorption, cell adhesion molecules, ABC transporters, glycosphingolipid biosynthesis-lacto and neolacto series, focal adhesion, glycosphingolipid biosynthesis-ganglio series, complement and coagulation cascades, cholesterol metabolism, biosynthesis of unsaturated fatty acids, glycerophospholipid metabolism, lysine degradation, and glycosphingolipid biosynthesis-globo and isoglobo series (Figure 5).

### 3.8 qRT-PCR validation

**Figure 6:**
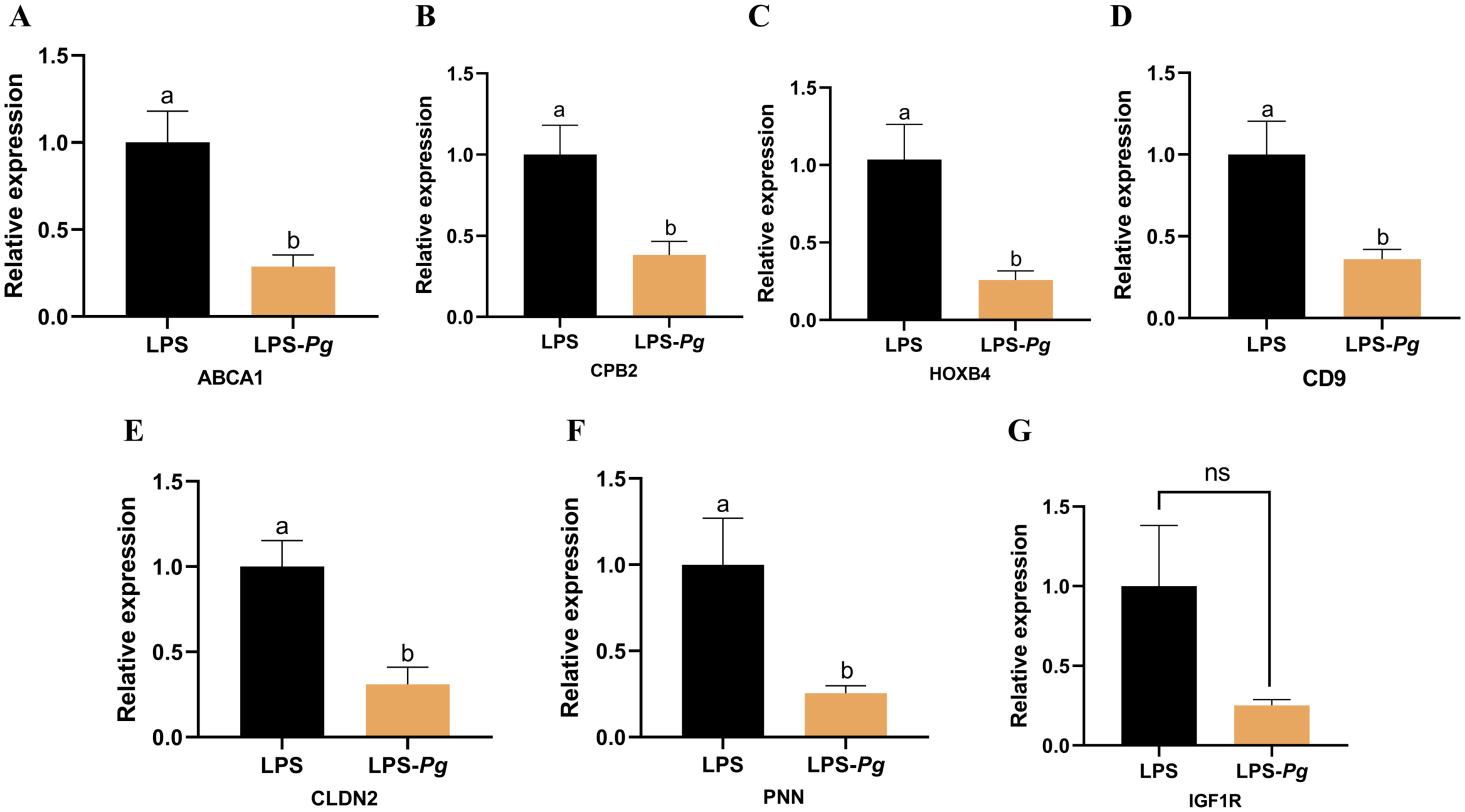
The DEGs were detected by qRT-PCR. **(A)** ABCA1. **(B)** CPB2. **(C)** HOXB4. **(D)** CD9. **(E)** CLDN2. **(F)** PNN. **(G)** IGF1R. Results are presented as mean ± SEM, significant differences (*p* < 0.05) are denoted by the distinct letters (a, b); unsignificant differences (*p* > 0.05) are denoted by the ns.

Eight DEGs were selected for qRT-PCR validation of RNA-Seq results. The expression levels of the seven genes were consistent with the sequencing data (Figure 6). The results indicate that RNA-seq was highly reliable.

## 4 Discussion

Parabacteroides are a newly identified class of probiotic bacteria, which are found in the intestinal tracts of animals. Their metabolites include acetic, propionic, and butyric acids, which are produced from fermented carbohydrates and are beneficial to host health (18). PG, a member of the Parabacteroides, exerts an anti-inflammatory effect in cells. Cell viability is an important indicator used to evaluate drug efficacy (19). In this study, the administration of the PG supernatant significantly increased the viability of IPEC-J2 cells. Tong et al. previously showed that the activity and growth parameters of IPEC-J2 cells gradually improved with increasing concentrations (ranging between 2 and 10 mg/mL) of *Bacillus amyloliquefaciens* J and *Lactiplantibacillus plantarum* SN4 using solid-state fermentation (20).

LPS, commonly referred to as an endotoxin, is an integral part of the outer cell membrane of Gram-negative bacteria (21). In the context of IPEC-J2 cells, LPS primarily acts as an inducer of inflammation and apoptosis and compromises the integrity of the damaged intestinal epithelium (22). Cytokines are pivotal mediators of the intestinal mucosal immune response during acute and chronic intestinal inflammation (23). In particular, interleukins (ILs) play crucial roles in cell-to-cell transmission, activation, and immunomodulation (24). Cytokines also mediate the activation, proliferation, and differentiation of T and B cells, and are closely associated with inflammatory responses. Existing research has demonstrated that upon stimulation by either IL-1β or LPS, the level of IL-6 secreted by enteric glial cells in the intestines of adult mice increases (25). LPS induces pro-inflammatory properties and increases the cellular IL-6 and IL-8 levels. Our study shows that in comparison with the control group, 1 μg/mL LPS can effectively increase the IL-6 and IL-8 levels in IPEC-J2 cells, indicating the induction of inflammatory damage and establishment of the inflammatory model.

Inflammation is a defense mechanism of the body that involves the migration of white blood cells to damaged tissues and the production of various cytokines, including IL-6, IL-1β, and IL-10. IL-6 is a pleiotropic proinflammatory cytokine that exhibits a wide range of functions (26, 27) and is rapidly produced due to body stress, tumorigenesis, and acute inflammatory responses. Thus, IL-6 plays a role in regulating immune responses and the acute-phase reaction. IL-8 is a chemokine that is produced by various cells in response to inflammatory stimuli. The recruitment and activation of neutrophils enables them to infiltrate the inflamed site (28), thereby exacerbating the inflammatory response, and thus, they are key to many inflammatory diseases such as rheumatoid arthritis (29) and inflammatory bowel disease (30). IL-10 can maintain the integrity of the tissue epithelium and promote the healing of damaged tissues (31). It can inhibit proinflammatory responses, reduce tissue damage, and exert anti-inflammatory effects. IL-10 is a major cytokine involved in intestinal regulation. The results of this study showed that treatment of IPEC-J2 cells with the culture supernatant from PG cells decreased the levels of the pro-inflammatory factor IL-6 and increased the levels of the anti-inflammatory factor IL-10. This indicates that the PG culture supernatant has a protective effect on cells induced for inflammation using LPS and plays a key role in the repair of damaged cells involved in cellular immune regulation. Similarly, Lai et al. found that PG antagonizes the activation of the TLR4/MD-2 signaling pathway, which participates in LPS-induced inflammation in gastric epithelial cells in the *Helicobacter pylori* infection model. As an emerging next-generation probiotic, PG can reduce the levels of pro-inflammatory cytokines IL-1β and TNF-*α* in mice with colitis, alleviate inflammatory damage, and maintain the integrity of the intestinal epithelium (43).

RNA-Seq was conducted to analyze the inflammatory mechanism underlying the effect of the PG culture supernatant on LPS-induced IPEC-J2 cells. The results showed that 36 genes were upregulated in the LPS-PG group and 2090 genes were downregulated. Specifically, genes related to inflammation and immunity, such as IL-6, IL-6R, and nuclear factor-kappa B1 (NF-κB1), were significantly downregulated in the inflammatory model group with the addition of the PG culture supernatant. IL-6 and IL-6R play crucial roles in the physiological and pathological processes of the body (32). Whereas NF-κB is a protein complex that plays a crucial role in biological processes (33). Cytokine production regulates the expression of target genes, prompting the cells to synthesize and release various cytokines. These cytokines play important roles in processes such as immune regulation and inflammatory responses. Furthermore, the unregulated gene Cytochrome b is a core component of the mitochondrial respiratory chain and plays a pivotal role in the cellular energy metabolic network, providing a sustained energy supply for the efficient operation of immune cells. This energy support mechanism not only maintains the basic functions of immune cells but also significantly enhances the body’s immune defense efficiency by strengthening the phagocytic activity, signal transduction, and cytokine secretion capacity. Thus, the PG supernatant can ameliorate the inflammatory response in IPEC-J2 cells via immune-related gene regulation.

GO enrichment analysis showed that the function for BP enrichment included cellular processes, the function with the most genes enriched in the CC category was the cellular anatomical entity, and the function with the most genes in the molecular function category was binding. Among them, the “Antioxidant activity” pathway is important for immune responses. A close and complex relationship exists between the antioxidant activity levels of the body and the immune system as they influence and regulate each other to jointly maintain the health of the body (34, 35). KEGG analysis showed that the PI3K-AKT pathway included 62 DEGs that are significantly enriched. The PI3K/Akt signaling pathway is crucial for immune regulation and the inflammatory response (36, 37). Most of the genes were related to immune function, among which IL-6R, NF-ĸB1, VEGFA, the integrin family, laminin family, collagen family, FN1, fibroblast growth factors and their receptor, EPHA2, and ERBB2 played major roles. In the development pathways associated with immune cells, PI3K-AKT is involved in the differentiation of T cells in the thymus and maturation of B cells in the bone marrow, laying the foundation for the construction of a sound immune system (38–40). In macrophages, after recognition of pathogen-associated molecular patterns through pattern-recognition receptors, the PI3K-AKT pathway is activated, enhancing the phagocytic ability and antigen-presenting efficiency and effectively initiating an immune response (41, 42). In addition, the PI3K-AKT pathway is involved in regulating the metabolism of immune cells to provide sufficient energy for their activation and function (39). These results suggested that the PG improve the inflammatory response of IPEC-induced IPEC-J2 cell by activated the PI3K-AKT pathway.

## 5 Conclusion

PG reduced the secretion of the pro-inflammatory cytokine IL-6 in LPS-induced IPEC-J2 cells and increased the secretion of the anti-inflammatory cytokine IL-10, thereby exerting anti-inflammatory effects *in vitro*. Transcriptomic analysis suggested that PG may mitigate LPS-induced immune damage in IPEC-J2 cells by modulating the PI3K-AKT pathway. Thus, *P. goldsteinii* is a potential probiotic for piglets. However, several key factors, such as interactions with the gut microbiota, host immune system responses, probiotic survival in the digestive tract, and long-term effects, are not captured in this model. In the future work, the Dextran Sulfate Sodium Salt -induced piglets will used to investigate the effect of *P. goldsteini in vivo* study.

## Data Availability

The raw sequencing data have been deposited in the China National GeneBank Sequence Archive (CNSA) of the China National GeneBank DataBase (CNGBdb) with the accession number CNP0005800.

## Footnotes

^1^ http://www.usadellab.org/cms/index.php?page=trimmomatic

^2^ https://ccb.jhu.edu/software/hisat2/index.shtml

^3^ http://www.Geneontology.org/

^4^ https://wego.genomics.cn/

^5^ https://www.ncbi.nlm.nih.gov/tools/primer-blast/
